# Letter to the Editor: New IUPAC guidelines for the reporting of stable hydrogen, carbon, and oxygen isotope-ratio data

**DOI:** 10.6028/jres.100.021

**Published:** 1995

**Authors:** Tyler B. Coplen

**Affiliations:** Secretary, IUPAC Commission on Atomic Weights and Isotopic Abundances, United States Geological Survey, 431 National Center, 12201 Sunrise Valley Drive, Reston, Virginia 22092, USA

On behalf of the International Union of Pure and Applied Chemistry (IUPAC), and in particular its Commission on Atomic Weights and Isotopic Abundances, I should like to inform the readers of the *Journal of Research of the National Institute of Standards and Technology* of the following, based on Refs. [1] and [2].

To eliminate possible confusion in the reporting of isotopic abundances on noncorresponding scales, the Commission on Atomic Weights and Isotopic Abundances of the International Union of Pure and Applied Chemistry (IUPAC) recommended at the IUPAC 37th General Assembly in August 1993 at Lisbon, Portugal, that (i) ^2^H/^1^H relative ratios of all substances be expressed relative to VSMOW (Vienna Standard Mean Ocean Water) on a scale such that ^2^H/^1^H of SLAP (Standard Light Antarctic Precipitation) is 0.572 times that of VSMOW; (ii) ^13^C/^12^C relative ratios of all substances be expressed relative to VPDB (Vienna Peedee belemnite) on a scale such that ^13^C/^12^C of NBS19-limestone (RM 8544) is 1.00195 times that of VPDB; and (iii) ^18^O/^16^O ratios of all substances be expressed relative to either VSMOW or VPDB on scales such that ^18^O/^16^O of SLAP is 0.9445 times that of VSMOW.

As a consequence, the Commission recommends that researchers should:

1. discontinue reporting isotopic abundances relative to SMOW (Standard Mean Ocean Water) and PDB (Peedee belemnite);
2. express hydrogen isotopic ratios of all substances relative to VSMOW (Vienna Standard Mean Ocean Water; RM 8535) on a normalized scale such that the δ^2^H of SLAP (Standard Light Antarctic Precipitation; RM 8537) relative to VS-MOW is −0.428;
3. express carbon isotopic ratios of all substances relative to VPDB (Vienna Peedee belemnite) on a scale defined by adopting a δ^13^C value of +0.001 95 for NBS19-limestone (RM 8544) relative to VPDB;
4. express oxygen isotopic ratios relative to either VSMOW or VPDB on a normalized scale such that the δ^18^O of SLAP (RM 8537) is −0.0555 relative to VSMOW (RM 8535); and
5. indicate values of isotopic fractionation factors if isotopic abundance measurements of minerals or compounds depend upon such factors.
